# Genomic copy number variation correlates with survival outcomes in WHO grade IV glioma

**DOI:** 10.1038/s41598-020-63789-9

**Published:** 2020-04-30

**Authors:** Zachary S. Buchwald, Sibo Tian, Michael Rossi, Geoffrey H. Smith, Jeffrey Switchenko, Jennifer E. Hauenstein, Carlos S. Moreno, Robert H. Press, Roshan S. Prabhu, Jim Zhong, Debra F. Saxe, Stewart G. Neill, Jeffrey J. Olson, Ian R. Crocker, Walter J. Curran, Hui-Kuo G. Shu

**Affiliations:** 1 Department of Radiation Oncology, Winship Cancer Institute, Emory University, Atlanta, GA USA; 2 Sema4 Genomics, Branford, CT USA; 3 Pathology & Laboratory Medicine, Emory University, Atlanta, GA USA; 4 Department of Biostatistics and Bioinformatics, Rollins School of Public Health, Emory University, Atlanta, GA USA; 5 Southeast Radiation Oncology Group, Levine Cancer Institute, Carolinas Healthcare System, Charlotte, NC USA; 6 Department of Neurosurgery, Winship Cancer Institute, Emory University, Atlanta, GA USA

**Keywords:** Genetics research, Cancer genomics, CNS cancer

## Abstract

Allele-specific copy number analysis of tumors (ASCAT) assesses copy number variations (CNV) while accounting for aberrant cell fraction and tumor ploidy. We evaluated if ASCAT**-**assessed CNV are associated with survival outcomes in 56 patients with WHO grade IV gliomas. Tumor data analyzed by Affymetrix OncoScan FFPE Assay yielded the log ratio (R) and B-allele frequency (BAF). Input into ASCAT quantified CNV using the segmentation function to measure copy number inflection points throughout the genome. Quantified CNV was reported as log R and BAF segment counts. Results were confirmed on The Cancer Genome Atlas (TCGA) glioblastoma dataset. 25 (44.6%) patients had MGMT hyper-methylated tumors, 6 (10.7%) were IDH1 mutated. Median follow-up was 36.4 months. Higher log R segment counts were associate with longer progression-free survival (PFS) [hazard ratio (HR) 0.32, p < 0.001], and overall survival (OS) [HR 0.45, p = 0.01], and was an independent predictor of PFS and OS on multivariable analysis. Higher BAF segment counts were linked to longer PFS (HR 0.49, p = 0.022) and OS (HR 0.49, p = 0.052). In the TCGA confirmation cohort, longer 12-month OS was seen in patients with higher BAF segment counts (62.3% vs. 51.9%, p = 0.0129) and higher log R (63.6% vs. 55.2%, p = 0.0696). Genomic CNV may be a novel prognostic biomarker for WHO grade IV glioma patient outcomes.

## Introduction

Glioblastoma is the most common intracranial malignancy with an age-adjusted incidence of 0.59 to 3.69 per 100,000 persons^1^. The prognosis is dismal with median survival ranging from 15–22 months^2,3^. The standard of care treatment, for patients with good performance status is maximal total resection followed by combination temozolomide and radiotherapy with or without NovoTTF^4^. For some patients, treatment can significantly impact their quality of life while conferring only marginal clinical benefit.

Molecular genetics has become important in many malignancies, the study of which have yielded both prognostic and/or predictive biomarkers. In glioma, mutations and epigenetic changes such as isocitrate dehydrogenase (IDH) mutations, chromosome 1p/19q co-deletion, and O^6^-methylguanine–DNA methyltransferase (MGMT) hypermethylation have demonstrated clinical importance. The role and benefit of adjuvant radiotherapy and temozolomide have been aided by the predictive biomarkers in grade II-IV gliomas^4–6^. For grade III glioma patients with an IDH1-mutantion or 1p/19q co-deletion, chemoradiotherapy significantly improves survival compared to radiotherapy alone^6,7^.

Genome-wide copy number analysis, an approach that assesses the number of gains and loses tiled across the genome, has been utilized with increasing frequency in a number of malignancies^8–11^. With the development of high-density single nucleotide polymorphism (SNP) microarrays, including OncoScan, greater robustness with higher resolution and the ability to detect copy number variations (CNV), including loss of heterozygosity, uniparental disomy, submicroscopic losses and gains, are possible^12^. Accurate quantification of tumor copy number gains and losses when tumors frequently deviate from a diploid state and populations of non-tumor cells are present within the tumor sample, however, has remained a persistent issue^13,14^. To overcome these challenges, a novel allele-specific copy number analysis of tumors (ASCAT) was developed by Loo *et al*. which can obtain accurate genome-wide ASCAT profiles while accounting for aberrant tumor cell fraction (non-tumor components), ploidy, gains, losses, loss of heterozygosity and copy number-neutral events^15^.

In glioblastoma, both TCGA and institutional data have been interrogated for CNV to evaluate different genes involved in oncogenesis and tumor behavior^16–18^. To our knowledge, genome-wide copy number analysis has not been correlated with clinical outcomes. Here, using data from the OncoScan SNP array, we applied the ASCAT algorithm to assess the impact of differences in ploidy, aberrant cell fraction (ACF), and CNV on progression-free survival (PFS) and overall survival (OS). We also generated a metric to assess the extent of copy number variation seen in the genome, a measure of overall genomic disorder, and correlated this with survival outcomes.

## Results

### Patient and treatment characteristics

56 patients who were eligible for analysis by ASCAT, and had a minimum follow-up of 4 months, were identified. This institutional cohort consisted of 22 females and 34 males. Median follow-up time was 36.4 months (range 4.6–42). The median age was 60.4 years (range: 26.1–86.3). The median KPS was 80 (range: 40–100). The extent of surgery was: 35 subtotal resections, 14 gross total resections, 6 biopsies and 1 unknown. Tumors were at least partially methylated at MGMT in 25 patients (44.6%) and harbored IDH1 mutations in 6 patients (10.7%).

44 patients received radiotherapy of at least 60 Gy, typically using volumetric arc therapy (VMAT) per institutional standard; other patients received treatment to less than 60 Gy due to either advanced age, poor performance status, physiologic decline, or received radiotherapy at an outside facility. 44 (78.5%) patients received concurrent temozolomide, and 38 (67.8%) patients received at least 1 cycle of adjuvant chemotherapy (37 of 38 received temozolomide and one patient received irinotecan). A full summary of patient and tumor characteristics can be found in Table 1.

**Table 1:** Summary of Patient, Tumor, and Treatment Characteristics.

Variable	Level	N = 56 (%)
**Patient Characteristics**		
Age (years)	Median (range)	60.4 (26.1-86.3)
Gender	Male	34 (60.7%)
	Female	22 (39.3%)
KPS	≤ 70	23 (42.6%)
	> 70	31 (57.4%)
Surgery	Biopsy	6 (10.9%)
	GTR	14 (25.5%)
	STR	35 (63.6%)
Radiotherapy	≥ 60 Gy	44 (86.2%)
	< 60 Gy	7 (13.7%)
Concurrent temozolomide	Yes	44 (78.5%)
	No	5 (8.9%)
	Unknown	7 (12.5%)
Adjuvant chemotherapy	Yes	38 (67.8%)
	No	8 (14.3%)
	Unknown	10 (17.8%)
**Tumor Characteristics**		
MGMT methylation	Unmethylated	31 (55.4%)
	Methylated/partially methylated	25 (44.6%)
IDH1 mutation	Negative	50 (89.3%)
	Positive	6 (10.7%)
1p19q co-deletion	No	51 (91.1%)
	Yes	4 (7.1%)
	Unknown	1 (1.8%)
ASCAT ploidy (numeric)	Median (SD)	2.05 (0.55)
	Range	(1.64-4.05)
ASCAT log R segment counts (numeric)	Median (SD)	371 (92.23)
	Range	(233-646)
ASCAT BAF segment counts (numeric)	Median (SD)	266 (96.93)
	Range	(156-572)
ASCAT ACF (numeric)	Median (SD)	0.78 (0.55)
	Range	(0.29-1)

Abbreviations: GTR, gross total resection; STR, subtotal resection; KPS, Karnofsky performance status; ASCAT, allele-specific copy number analysis of tumors; BAF, B-allele frequency; ACF, aberrant cell fraction; log R, log ratio.

For the entire cohort, median PFS was 8 months; 12- and 24-month PFS were 24.1% and 9.4%, respectively. Median OS was 14.7 months; 12- and 24-month OS were 59.8% and 22.4%, respectively. On univariate analysis, patients with IDH1 mutations (HR 0.29, p = 0.022) and those with 1p19q co-deletions (HR 0.29, p = 0.046) had longer PFS (Table S1). Higher KPS (>70 vs ≤70, p = 0.011), greater extent of resection (biopsy vs. STR, p = 0.005) and MGMT methylation status (p = 0.002) were statistically significant for longer OS (Table S1).

### ASCAT Biomarkers and clinical outcomes

**Figure 1:**
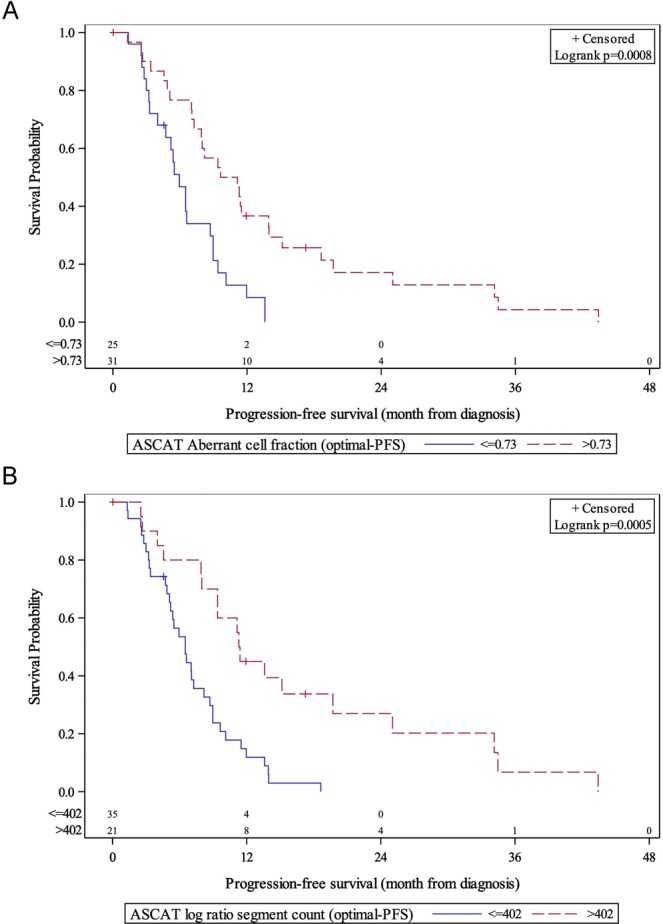
PFS stratified by ASCAT aberrant cell fraction (**A**) and log R segment counts **(B**). χ^2^ = 11.27 (A), χ^2^ = 12.31 (B); degree of freedom = 1 (A,B); total N = 56.

**Figure 2:**
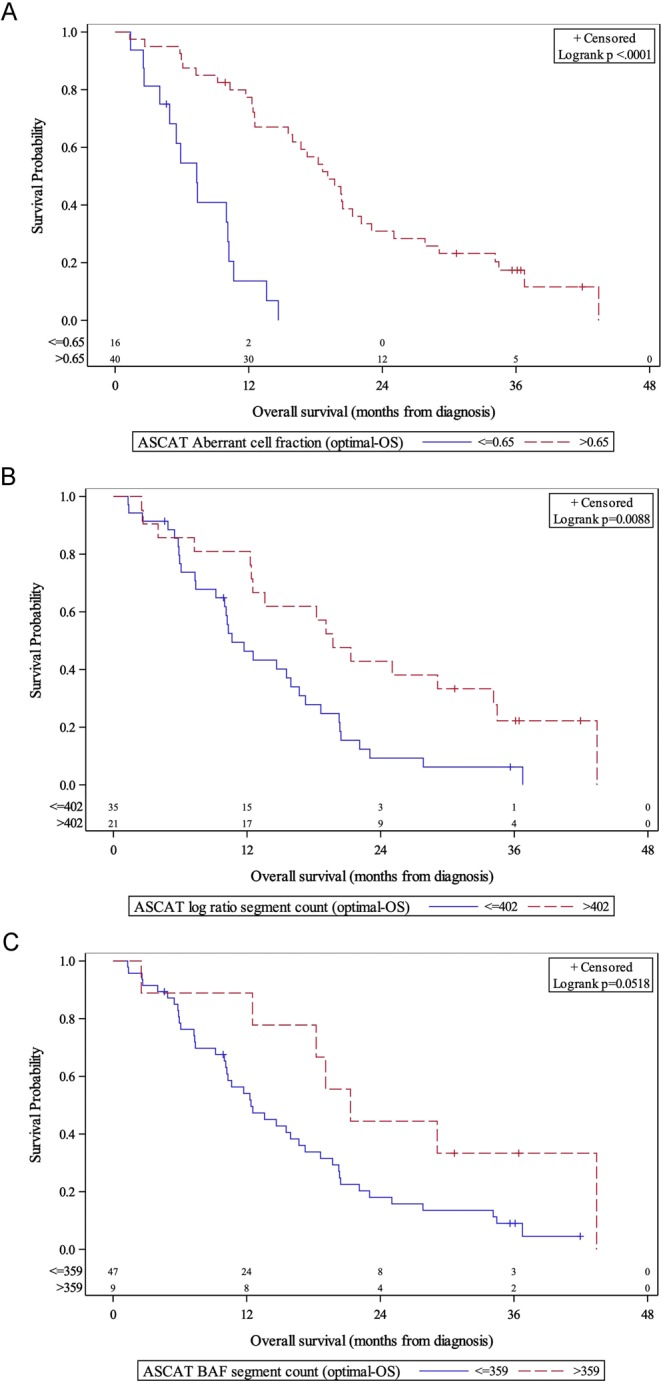
OS stratified by ASCAT aberrant cell fraction (**A**) and log R segment counts (**B**), and B-allele frequency (**C**). χ^2^ = 30.44 (A), χ^2^ = 6.86 (B), χ^2^ = 3.78 (C); degree of freedom = 1 (A–C); total N = 56.

All 56 patients had tissue analyzed with the OncoScan SNP Array. We first evaluated each ASCAT parameter independently. We found that patients with higher ACF (malignant cell fraction) demonstrated longer 12-month PFS (36.7% vs 8.5*%*, p = 0.0008) (Fig. 1A). We next evaluated and quantified the number of copy number inflection points in the genome using the ASCAT segmentation function. This was defined as the segment counts. Patients with higher log ratio (R) segment counts had longer 12-month PFS (45.0% vs 11.9%, p = 0.0005) (Fig. 1B). Patients with higher ACF (77.3% vs 13.6%, p < 0.0001) and higher log R segment counts (81% vs 46.3%, p = 0.0088) had longer 12-month OS (Fig. 2A,B). 12-month OS, as stratified by BAF segment counts (88.9% vs 54.1%), trended towards significance (p = 0.0518) (Fig. 2C).

We next performed a univariate analysis, which confirmed elevated ASCAT ACF (hazard ratio [HR] 0.36, 95% confidence interval [CI] 0.2–0.67, >0.73 vs ≤0.73, p = 0.001), elevated log R segment counts (HR 0.32 (95% CI 0.17–0.63), (>402 vs. ≤402, p < 0.001), and elevated BAF segment counts (HR 0.49 (95% CI 0.27–0.90), >277 vs. ≤277, p = 0.022) were all associated with longer PFS (Table S1). On univariate analysis of OS, those with higher ACF (HR 0.14 (95% CI 0.07–0.31), >0.65 vs. ≤0.65, p < 0.001), higher log R segment counts (HR 0.45 (95% CI 0.24–0.83), >402 vs. ≤402, p = 0.01) had significantly better OS. Patients with higher BAF segment counts had nearly significant better OS (HR 0.49 (95% CI 0.29–0.9), >359 vs ≤359, p = 0.052). These findings demonstrate that in our institutional cohort a higher tumor component (aberrant cells), and greater CNV correlate with improved clinical outcomes.

To evaluate whether our ASCAT parameters were independent of known clinical risk factors, we evaluated a total of five variables (IDH1 mutations status, 1p19q co-deletion status, MGMT methylation status, KPS, and ASCAT parameter) in each multivariable PFS model due to limited sample size. On multivariable analysis of PFS, both higher ASCAT ACF (HR 0.44 (95% CI 0.22–0.88), >0.73 vs. ≤0.73, p = 0.021) and ASCAT log R segment counts (HR 0.31 (95% CI 0.14–0.68), >402 vs. ≤402, p = 0.004) remained statistically significant (Table 2). A trend was observed between higher BAF segment counts and PFS (HR 0.54 (95% CI 0.27–1.08), >277 vs. ≤277, p = 0.079). For OS, the multivariate analysis included IDH1 mutations status, MGMT methylation status, extent of resection, KPS, and ASCAT parameter. ASCAT ACF (HR 0.26 (95% CI 0.10–0.7), >0.65 vs. ≤0.65, p = 0.007) and ASCAT log R segment counts (HR 0.42 (95% CI 0.19–0.91), >402 vs. ≤402, p = 0.027) remained statistically significant (Table 3). These results confirm that both increased ACF and increased log R segment counts (a reflection of higher CNV) are independently clinically prognostic.

**Table 2:** Multivariable analysis of PFS.

Covariate	Level	Hazard Ratio (95% CI)	P-value
ASCAT ACF	>0.73	0.44 (0.22 – 0.88)	**0.021**
	≤0.73	—	
IDH1 mutated	Yes	0.29 (0.03 - 3.06)	0.304
	No	—	
MGMT	Meth	0.92 (0.48-1.77)	0.813
	Unmeth	—	
KPS	>70	0.99 (0.54-1.84)	0.984
	≤70		
1p19q co-deleted	Yes	1.07 (0.1-11.02)	0.957
	No		
**Covariate**	**Level**	**Hazard Ratio (95% CI)**	**P-value**
ASCAT log R seg. counts	>402	0.31 (0.14 – 0.68)	**0.004**
	≤402	—	
IDH1 mutated	Yes	0.14 (0.01 – 2.6)	0.190
	No	—	
MGMT	Meth	1.15 (0.59-2.25)	0.683
	Unmeth	—	
KPS	>70	0.86 (0.47-1.58)	0.632
	≤70		
1p19q co-deleted	Yes	2.17 (0.12-39.49)	0.600
	No		
**Covariate**	**Level**	**Hazard Ratio (95% CI)**	**P-value**
ASCAT BAF seg. counts	>277	0.54 (0.27 – 1.08)	0.079
	≤277	—	
IDH1 mutated	Yes	0.16 (0.01 – 2.22)	0.173
	No	—	
MGMT	Meth	0.99 (0.50-1.96)	0.975
	Unmeth	—	
KPS	>70	0.82 (0.45-1.49)	0.508
	≤70		
1p19q co-deleted	Yes	1.81 (0.13-25.06)	0.658
	No		

Abbreviations: KPS, Karnofsky performance status; ASCAT, allele-specific copy number analysis of tumors; BAF, B-allele frequency; ACF, aberrant cell fraction; log R, log ratio; CI, confidence interval; PFS, progression free survival.

**Table 3:** Multivariable analysis of OS.

Covariate	Level	Hazard Ratio (95% CI)	P-value
ASCAT ACF	>0.65	0.26 (0.10 – 0.7)	**0.007**
	≤0.65	—	
IDH1 mutated	Yes	0.56 (0.18 – 1.76)	0.324
	No	—	
MGMT	Meth	0.61 (0.29-1.29)	0.200
	Unmeth	—	
KPS	>70	0.81 (0.36-1.79)	0.596
	≤70	—	
Surgery Type	Biopsy	1.78 (0.67-4.76)	0.248
	GTR	1.13 (0.54-2.38)	0.749
	STR	—	
**Covariate**	**Level**	**Hazard Ratio (95% CI)**	**P-value**
ASCAT log R seg. counts	>402	0.42 (0.19 – 0.91)	**0.027**
	≤402	—	
IDH1 mutated	Yes	0.61 (0.19 – 1.95)	0.406
	No	—	
MGMT	Meth	0.69 (0.33-1.41)	0.303
	Unmeth	—	
KPS	>70	0.51 (0.25-1.01)	0.504
	≤70	—	
Surgery Type	Biopsy	3.69 (1.26-10.79)	**0.017**
	GTR	1.04 (0.50-2.17)	0.912
	STR	—	

Abbreviations: GTR, gross total resection; STR, subtotal resection; KPS, Karnofsky performance status; ASCAT, allele-specific copy number analysis of tumors; ACF, aberrant cell fraction; log R, log ratio; CI, confidence interval; OS, overall survival.

### TCGA Cohort

**Figure 3:**
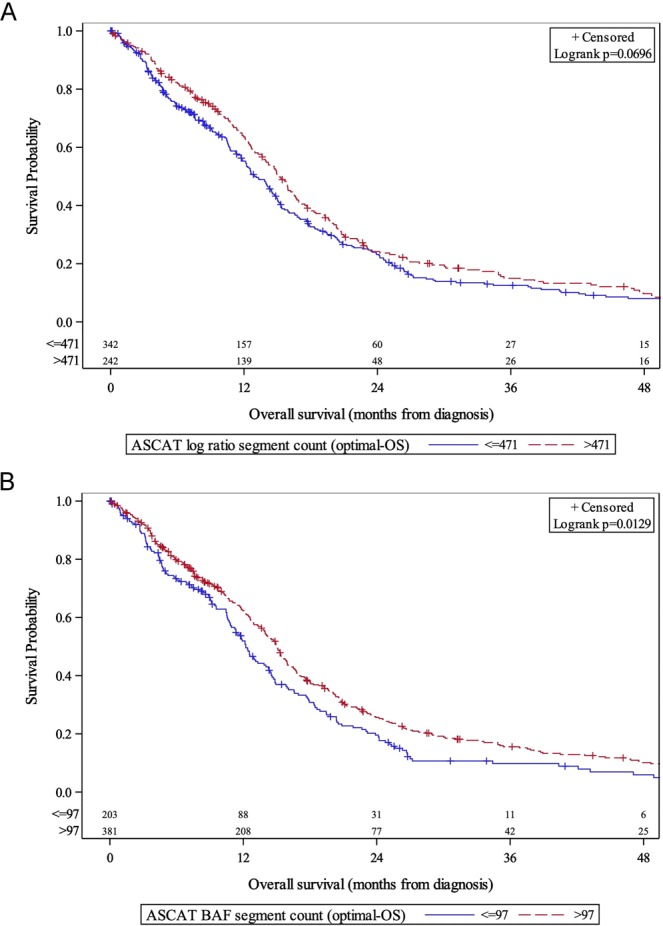
OS stratified by TCGA-derived ASCAT log R segment counts (**A**) and B-allele frequency (**B**). χ^2^ = 3.29 (A), χ^2^ = 6.18 (B); degree of freedom = 1 (A,B); total N = 584.

To assess the validity of our assay and methodology, an independent cohort, TCGA, was utilized. This dataset has comprehensive genomics data with patient survival outcomes. Multiple SNP arrays were utilized for the GBM cohort. Of the ones used, Affymetrix Genome-Wide Human SNP Array 6.0 (Affy SNP 6) was compatible with ASCAT. The Affy SNP 6 array has different probe numbers and locations, and therefore OS cut-points utilized for the Oncoscan dataset could not be applied for this analysis. A total of 564 patients’ tumor data were analyzed. Utilizing optimal cut-points for TCGA, higher BAF segment counts (>97 vs. ≤97) were associated with longer 12-month OS (62.3% vs. 51.9%, p = 0.0129). A trend towards significance was observed for log R segment counts (>471 vs. ≤471), 12-month OS was 63.6% vs. 55.2% (p = 0.0696) (Fig. 3A,B). A multi-variable analysis of OS, including optimal cutpoint BAF segment counts, as well as age and race revealed that BAF segment counts remained significant as a predictor of OS (HR 0.81, p = 0.034) [Table 4]. A summary of survival endpoints at 12- and 24-months by ASCAT variable, optimal cut-point, for the institutional and TCGA validation cohorts is shown in Table S2.

**Table 4:** Multivariable analysis of OS in TCGA validation cohort.

Covariate	Level	Hazard Ratio (95% CI)	P-value
ASCAT BAF seg. counts	>97	0.81 (0.67 – 0.98)	**0.034**
	≤97	—	
Race	Asian	0.64 (0.30-1.37)	0.252
	Black	0.84 (0.59-1.20)	0.343
	White	—	
Age		1.03 (1.03-1.04)	**<0.001**

Abbreviations: ASCAT, allele-specific copy number analysis of tumors; BAF, B-allele frequency.

## Discussion

Appropriate patient selection for aggressive treatment strategies, and corresponding patient counseling are of paramount importance. Highly toxic treatments that confer little, if any, clinical benefit, but come at significant quality of life costs, should be avoided. A number of important molecular biomarkers that are prognostic and/or predictive have already been identified in glioma patients including 1p19q co-deletion, IDH1 mutation, and MGMT methylation. Genome-wide level analyses can be complementary to these biomarkers, and may provide additional information on outcomes.

In this study, we demonstrated that higher whole-genome CNV correlates with longer survival outcomes on both our institutional and validation cohorts. Of note, due to differences in total probe number and location within the genome, the two input SNP arrays, Oncoscan (institutional data set) and Affymetrix SNP 6 (TCGA data set), could not be directly compared using the same ASCAT cut-points for the different parameters. Therefore, for future assay utilization, it is likely that the cut-point will be identified for the individual SNP array utilized. However, once these assay specific cut-points are established, we believe the clinical utility is clear. Additionally, despite these large differences between the two assays, they both demonstrated longer survival for increased CNV. This suggests a deeper biological significance, clear value in utilizing this validation cohort, and it warrants further investigation.

In contrast to our study, CNV has also been evaluated in a number of other malignancies and a higher CNV burden is most commonly associated with worse outcomes^19,20^. CNV has also been shown to be important in increasing cancer risk and accelerating disease progression^21–23^. In this context, our results are intriguing. Interestingly, in the context of glioblastoma, a reduction in copy number segments of specific genes confer a shorter survival^24^. Lehrer *et al*. showed a longer median survival for patients with increased copy number segments of SGK1, a gene required for growth and survival of glioblastoma stem-cells^24^. Others have shown the converse, that total CNV was shown to be a prognostic factor for worse outcomes in adult astrocytoma, especially in the IDH-mut group^25^. In the context of our study, this subgroup represented a very small fraction of the patient samples we evaluated and may therefore not be directly applicable. Others have also showna strong correlation between CNV and shorter disease-free and overall survival^26^. It remains interesting that our study stands in contrast to the work of a few other groups. However, the clinical management of patients in the other studies was often not well described. It is possible that higher CNV reflects a poor prognostic factor in the absence of optimal therapy, but similar to Her2neu + breast cancer, it may serve as a predictive biomarker for benefit with specific therapy, in this case chemoradiation. This questions, unfortunately, could not be addressed in the current study.

The data concerning radiation sensitivity and CNV is mixed. Yard *et al*. showed that in irradiated colorectal, uterine and ovarian carcinoma human cell lines, there was a positive correlation with increased somatic copy number alterations and survival^27^. They argued that the same mechanism that generated the copy number alterations initially also increased a tumor cell’s capability to repair double-stranded breaks following radiotherapy. Other studies have shown that the sensitivity to radiation depends on gene dosage and, specifically, which gene copy numbers are altered. A reduction in Rad51 expression, for example, in glioma cells significantly increases radiosensitivity^28^. In this case, our results are hypothesis generating. It may be that the broad and extensive coverage of the genome with copy number variation tips the balance towards radiosensitivity, but further study is warranted.

The increased aberrant cell fraction (ACF) also correlated with both longer PFS (p = 0.001) and OS (p < 0.001) in our institutional dataset. The non-aberrant cells reflect the tumor microenvironment, and these results suggest that non-aberrant cells are either tumor promoting or are involved in treatment resistance. The non-tumor glioma microenvironment is a complex interplay of stem cells, astrocytes, and immune cells^29,30^. It has been shown that the accumulation of microglia/brain macrophages around the tumor correlates with poor clinical prognosis^31^. In addition, tumor infiltrating lymphocytes (TILs) have also demonstrated a correlation with survival. Han *et al*. showed using immunohistochemistry for CD4+ FoxP3+ and CD8+ TILs, there was an inverse correlation between tumor grade and the number of CD8+ TILs. Additionally, they showed that FoxP3+ T-cells (regulatory T-cells) were only observed in glioblastomas, and not in lower grade gliomas^32^. In glioblastoma patients, a high CD4 to CD8 ratio was a predictor of worse survival outcomes^32^. In contrast, another study demonstrated that higher levels of CD8+ TILs had shorter survival, however, this was confounded by the association between low CD8+ TILs and methylation of MGMT^33^. These data combined with our observations suggest that the worse outcomes with lower ACF may be a function of increased myeloid or lymphoid immunosuppressive infiltrates. These results also have implications for the on-going checkpoint blockade clinical trials in high-grade gliomas with interim results from CheckMate-143 (nivolumab versus bevacizumab for recurrent GBM) failing to show an OS benefitfor nivolumab^34^. It is possible that more myeloid-derived suppressor cells or FoxP3+ CD4+ T-cells are responsible for the worse outcomes observed in patients with lower ACF, and may impact responses to immunotherapy^35–37^. Notably, this idea is supported by a retrospective analysis of the nivolumab study which showed decreases in MDSCs in peripheral blood correlated with treatment response^37^.

Our study’s relatively small size and retrospective nature are inherent limitations. Additionally, due to the small sample size, we were not able to incorporate all potentially relevant variables into our multivariable analysis including radiation dose, timing of temozolomide administration, and use of other therapies such as NovoTTF. Use of a WHO grade IV glioma cohort represents some inherent heterogeneity. The molecular definition of WHO grade IV gliomas makes distinctions based on IDH1 mutation and 1p19q co-deletion status. Here, the proportion of patients with these 2 mutations account for a relatively small minority. The inclusion of IDH1 mutation and 1q19q co-deletion status in the multi-variable models take into account their contribution towards heterogeneity. Notably, 1p19q co-deletion status and IDH1 status were not significantly linked to PFS in the multi-variable model when ASCAT variables were present; and IDH1 mutation was similarly non-significant in the OS multi-variable model (which has substantial overlap with 1q19q co-deletion as a sub-population). Despite these limitations, the results remain thought provoking and warrant further validation and investigation.

## Methods

### Patients

Records of patients treated at a single academic institution between 1/2009 and 8/2015 were evaluated and reviewed; this was performed under a protocol approved by the Winship Cancer Institute institutional review board. Data were de-identified according to the Health Insurance Portability and Accountability Act, and all methods were performed in accordance with the relevant guidelines and regulations. Informed consent was obtained for tissue sample banking; informed consent for this study was waived by the Institutional Review Board that approved this study’s protocols. Inclusion criteria included a pathologic diagnosis of a primary WHO grade IV glioma, no prior brain radiotherapy and an OncoScan analysis for ASCAT inputs.

### Treatment

Patients with newly-diagnosed WHO grade IV gliomas were discussed at a multi-disciplinary tumor board, including surgical oncology, neurosurgery, pathology, radiology, and medical and radiation oncology services. Decisions for therapeutic management were done jointly. Recorded baseline characteristics included age, gender, tumor location, performance status, MGMT methylation, 1p19q co-deletion and IDH1/2 mutation status. Radiation characteristics were also recorded, including radiation total dose, dose per fraction, and receipt of concurrent and adjuvant temozolomide.

### SNP array

For the primary (institutional) dataset, an Affymetrix OncoScan FFPE single nucleotide polymorphism (SNP) array was used for raw genomic data acquisition. Two data tracks were produced from the SNP array: total signal intensity and allelic contrast. Log ratio (R) represents the total signal intensity, which reflects the total copy number on a logarithmic scale. The B-allele frequency (BAF) represents the allelic contrast and demonstrates the relative presence at each SNP locus evaluated of the two alternative nucleotides. For The Cancer Genome Atlas (TCGA) dataset, Affymetrix Genome-Wide Human SNP Array 6.0 was utilized for the raw genomic data.

### ASCAT analysis

Following acquisition of the raw genomic data from biopsy or resected tumor samples, for each microarray case in Affymetrix Chromosome Analysis Suite v3.0, the Export Probe Level Data function was used to create a text file with the microarray probe set name, log 2 ratio signal, and BAF signal suitable for processing with the ASCAT v2.4.4 library in R v3.4.1. TCGA data set, for which the raw data was previously acquired with the Affymetrix Genome-Wide Human SNP Array 6.0, was processed using an R-package “Rawcopy” to create the probe level BAF and log R data.

These data were used as inputs of the ASCAT algorithm. As a function of the SNP data, ASCAT models the allele-specific copy number and arrives at the solution that is closest to integer copies at all assessed loci. To do this accurately, the ASCAT algorithm accounts for important factors including polyploidy and aberrant cell fraction when calculating the CNV profile^8^. Aneuploidy, a deviation from the normal chromosomal number, can confound copy number analysis. Non-aberrant cell admixture or aberrant cell fraction reflects the non-tumor component of the sample and can differ significantly between samples again confounding copy number studies. As an intermediate output of the ASCAT algorithm, we quantified the number of segments with changes in the log R and BAF. A segment is defined as a region of the genome with a fixed copy number. For a representative log R data sample, there were an average of 4,866,610 base pairs and 332 probes per segment with a range of 109,439–8,276,5227 base pairs and 7–5,997 probes. The total number of segments reflects the number of CNV inflection points or changes in copy number in the genome. This was defined as the segment counts.

### Statistical analysis

PFS was defined as time in months from diagnosis to progression, death or last follow-up, where those alive without progression were censored at last follow-up. Progression was defined radiographically based on the T1 post-contrast MRI, as assessed by post-treatment neuroradiology reports and supplemented with information from tumor board evaluation, specialized MR sequences, and tissue confirmation where available. OS was defined as time in months from diagnosis to death or last follow-up, where those who survived were censored at last follow-up. Genomic characteristics considered for analysis were ASCAT ploidy, ASCAT aberrant cell fraction, ASCAT log ratio segment counts, and ASCAT BAF segment counts. These variables were analyzed as continuous variables, and as categorical variables dichotomized at their median values. Cut point analyses were also performed to identify statistically significant cut points for each genomic variable and each endpoint using an outcome-oriented approach^38^. All survival data with stratification were reported using the optimal cut point. The log-rank statistic was maximized, and the significance of each cut point was assessed. Statistically significant cut points were considered for further analysis.

Descriptive statistics were reported for each variable. PFS and OS curves were estimated using the Kaplan-Meier method, and compared using log-rank tests. Median follow-up was estimated using the reverse Kaplan-Meier approach, where the censoring indicator is switched such that deaths are censored. Univariate Cox proportional hazards models were also fit, modeling PFS and OS as a function of age, gender, Karnofsky performance status (KPS), surgery type, the four genomic characteristics, IDH1 mutation (positive vs. negative), and MGMT (methylated vs. unmethylated). Multivariable Cox proportional hazards models were fit for OS and PFS as function of the statistically significant ASCAT variable, IDH1, MGMT, KPS and 1p19q co-deletion. Multivariable Cox models also were fit for OS as a function of the statistically significant ASCAT variables, IDH1, MGMT, KPS, and surgery type. Model assumptions were checked and verified. Statistical analyses were performed using SAS 9.4 (SAS Institute Inc., Cary, NC), and statistical significance was assessed at the 0.05 level.

### GEO

Accession number – GSE125255 https://www.ncbi.nlm.nih.gov/geo/query/acc.cgi?acc=GSE125255.

## Supplementary information

Supplementary information
