# Supplementary material for: Genomic copy number variation correlates with survival outcomes in WHO grade IV glioma
Source: Sci Rep. 2020 Apr 30;10:7355. doi: 10.1038/s41598-020-63789-9 (PMC7192941; doi:10.1038/s41598-020-63789-9)
Supplement: Supplementary file 1 — Supplementary information [file 41598_2020_63789_MOESM1_ESM.pdf]

Genomic copy number variation correlates with survival outcomes in WHO grade IV glioma

Running Title: Genomic copy number variation in WHO grade IV glioma

Zachary S. Buchwald, MD, PhD[1*#]
Sibo Tian, MD[1#]
Michael Rossi, PhD[2]
Geoffrey H. Smith, MD[3]
Jeffrey Switchenko, PhD[4]
Jennifer E. Hauenstein, MS[3]
Carlos S. Moreno, PhD[3]
Robert H. Press, MD[1]
Roshan S. Prabhu, MD[5]
Jim Zhong, MD[1]
Debra F. Saxe, MD[3]
Stewart G. Neill, MD[3]
Jeffrey J. Olson, MD[6]
Ian R. Crocker, MD[1]
Walter J. Curran, MD[1]
Hui-Kuo G. Shu, MD, PhD[1]

[1]Department of Radiation Oncology, Winship Cancer Institute, Emory University, Atlanta, GA
[2]Sema4 Genomics, Branford, CT
[3]Pathology & Laboratory Medicine, Emory University, Atlanta, GA
[4]Department of Biostatistics and Bioinformatics, Rollins School of Public Health, Emory University, Atlanta, GA
[5]Southeast Radiation Oncology Group, Levine Cancer Institute, Carolinas Healthcare System, Charlotte, NC
[6]Department of Neurosurgery, Winship Cancer Institute, Emory University, Atlanta, GA

***Corresponding Author:**
Zachary S. Buchwald, MD PhD
Department of Radiation Oncology
Winship Cancer Institute, Emory University
1365 Clifton Rd NE
Atlanta, GA 30322
Telephone: (404) 778-3763
Fax: (404) 778-4139
zbuchwa@emory.edu

#Co-first author

Table S1. Univariate analysis of PFS and OS

Progression Free Survival

Variable	Level	N	Hazard Ratio (95% CI)	P-value
KPS	>70	31	0.79 (0.424 – 1.4)	0.424
	≤70	23	-	
Surgery Type	Biopsy	6	1.96 (0.81 – 4.78)	0.137
	GTR	14	0.79 (0.41-1.53)	0.491
	STR	35	-	
IDH1 mutated	Yes	6	0.29 (.10-0.84)	**0.022**
	No	50	-	
MGMT	Meth	25	0.63 (0.35-1.13)	0.121
	Unmeth	31	-	
1p19q co-deleted	Yes	4	0.29 (0.09-0.98)	**0.046**
	No	51	-	
ASCAT log R seg. count	>402	21	0.32 (0.17-0.63)	**<0.001**
	≤402	35	-	
ASCAT BAF seg. count	>277	25	0.49 (0.27-0.9)	**<0.022**
	≤277	31		
ASCAT ACF	>0.73	31	0.36 (0.20-0.67)	**0.001**
	≤0.73	25		

Overall Survival

Variable	Level	N	Hazard Ratio (95% CI)	P-value
KPS	>70	31	0.46 (0.26 – 0.84)	**0.011**
	≤70	23	-	
Surgery Type	Biopsy	6	3.89 (1.52 – 9.94)	**0.005**
	GTR	14	0.94 (0.48-1.87)	0.867
	STR	35	-	

IDH1 mutated	Yes	6	0.41 (.15-1.14)	0.077
	No	50	--	
MGMT	Meth	25	0.38 (0.21-0.71)	**0.002**
	Unmeth	31	-	
1p19q co-deleted	Yes	4	0.56 (0.17-1.82)	0.335
	No	51	-	
ASCAT log R seg. count	>402	21	0.45 (0.24-0.83)	**0.010**
	≤402	35	-	
ASCAT BAF seg. count	>359	9	0.49 (0.27-0.9)	0.052
	≤359	47	-	
ASCAT ACF	>0.65	40	0.14 (0.07-0.31)	**<0.001**
	≤0.65	1	-	

Abbreviations: GTR, gross total resection; STR, subtotal resection; KPS, Karnofsky performance status; ASCAT, allele-specific copy number analysis of tumors; ACF, aberrant cell fraction; log R, log ratio; BAF, B-allele frequency; CI, confidence interval; PFS, progression free survival; OS, overall survival

Table S2. Summary of survival endpoints by ASCAT variables

Endpoint/cohort	Variable	Optimal cut-point	Median survival in months (95% CI)	12-month survival	24-month survival	Log-rank p-value
PFS/institutional	ACF	≤0.73	5.9 (4, 8.7)	8.5%	0%	**<0.001**
		>0.73	10.4 (7.3, 13.9)	36.7%	17.1%	
	Log R seg. count	≤402	6.5 (5.1, 8.2)	11.9%	0%	**<0.001**
		>402	11.4 (7.9, 19.7)	45%	27%	
OS/institutional	ACF	≤0.65	7.3 (4, 10.2)	13.6%	0%	**<0.001**
		>0.65	19.1 (15.5, 22.1)	77.3%	30.9%	
	Log R seg. count	≤402	10.6 (7.4, 15.9	46.3%	9.3%	**0.009**
		>402	19.7 (12.4, 34.1)	81%	42.9%	
	BAF seg. count	≤359	12.4 (10, 16.7)	54.1%	18%	0.052
		>359	21.3 (2.5, 43.4)	88.9%	44.4%	
OS/validation cohort	Log R seg. count	≤471	12.9 (11.8, 14.5)	55.2%	23.2%	0.070
		>471	15.1 (13.6, 16.5)	63.6%	24.2%	
	BAF seg. count	≤97	12.2 (10.8, 14.3)	51.9%	19.6%	**0.013**
		>97	15 (13.9, 15.9)	62.3%	25.6%	

Abbreviations: Log R, log ratio; ACF, aberrant cell fraction, BAF, B-allele frequency, CI, confidence interval; PFS, progression free survival; OS, overall survival
